# The impact of podcast-based interventions on mental health: A systematic scoping review

**DOI:** 10.1371/journal.pmen.0000272

**Published:** 2025-03-19

**Authors:** Elise R. Carrotte, Beth Hobern, Alsa Wu, Christopher Groot, Fincina Hopgood, Michelle Blanchard, Lisa Phillips

**Affiliations:** Instituto Federal do Maranhão: Instituto Federal de Educacao Ciencia e Tecnologia do Maranhão, BRAZIL

## Abstract

Podcasts are increasingly being used for mental health-related messaging. The objective of this scoping review is to understand how podcasts are being used as a platform for mental health-related interventions (‘podcast-based interventions’). Six databases were searched: CENTRAL, EMBASE, PsycINFO, Communication and Mass Media Complete, Web of Science, and ProQuest Dissertations & Theses Global. Journal articles, conference proceedings, and dissertations were eligible for inclusion. Eligible studies included an audio-only podcast-based condition with at least one quantitative mental health-related outcome, including symptoms, treatment or management of mental health issues, mental health literacy or knowledge, and mental illness stigma, prejudice or discrimination, and involved experimental and quasi-experimental designs. Databases were searched for English-language results up to August 4, 2024. Across all searches, there were 2958 records identified, with 2468 screened after removal of duplicates; 20 unique studies met inclusion criteria. Most common podcast intervention types were meditation or mindfulness exercises, comprising eight (40%) of the studies, and psychoeducational or therapeutic content, also comprising eight (40%) studies. Most podcasts were streamed or downloaded online, and interventions ranged 1-28 episodes. Studies typically involved university or general community convenience samples, and median attrition was 42% (*IQR* = 10-50%). Researchers most studied the impact of podcast-listening on anxiety, reported in 35% of studies, followed by depressive symptoms (30%), stress or psychological distress (25%), body image-related variables (20%), and stigma (20%). There is some evidence supporting the efficacy of podcast-based interventions on various mental health-related outcomes, including improvements in mindfulness, body image, and stigmatising attitudes. Results highlighted the breadth of experimental and quasi-experimental studies involving podcast-based interventions with mental health-related outcomes. These podcasts show promising mental health-related outcomes worthy of further study and refinement. Study generalisability was limited predominantly by self-report data, convenience samples, and high attrition rates.

## Introduction

The last two decades have seen an influx in Internet-based and digital mental health content. Such media offer unique opportunities to increase the availability and reach of mental health-related information and self-help content, often free or low-cost. For example, SMS-based mental health interventions have been used to target a range of outcomes including reducing symptoms of anxiety and depression, coping with suicidal ideation, and decreasing alcohol and cigarette use [1]. With the rise of the smartphone era, thousands of mental health-related smartphone applications have been released, with evidence supporting their effects on improving psychological symptoms and quality of life [2], as well as generally being perceived by users as acceptable and helpful [3]. Furthermore, as the COVID-19 global pandemic triggered a series of lockdowns from 2020 onwards, people have increasingly relied on digital mental health interventions in place of in-person supports, such as telehealth services, Internet-based support groups, and psychoeducation webinars [4].

Podcasts are another form of digital media with potential to positively impact upon mental health. Unlike traditional radio, a podcast is ‘a piece of episodical, downloadable or streamable, primarily spoken audio content, distributed via the internet, playable anywhere, at any time, produced by anyone who so wishes’ [5, p. 11]. Podcasts are typically distributed through an RSS (‘Really Simple Syndication’) Feed, which distributes the file to a range of streaming platforms [6]. As a result, podcast episodes can be streamed or downloaded to a computer or mobile device using distribution apps such as Apple Podcasts and Spotify. Podcasts are now extremely common, with an estimated four million podcasts series as of 2023 [7]. Uptake is also high, with around half of American and Australian adults listening to a podcast within the last month [7,8], and listeners reporting podcasts to be accessible, engaging, and diverse [9,10].

Podcasts are increasingly being used in the mental health field; like other mental health interventions [11], including media-based interventions, they may target the general public or whole population (i.e., as universal interventions). Or, at a smaller scale, they may target individuals who currently have, or are at risk of developing a mental health condition (i.e., selective or indicated interventions) [11]. According to podcast database ListenNotes, there are over 2000 podcast series publicly available with ‘mental health’ in the title [12]. Topics include psychoeducation (such as coping or self-help tips), marriage, couples and family relationship advice, and education and professional development aimed at mental health professionals [13,14]. Podcasts may feature hosts and guests with lived experience of mental health issues, mental health professionals, advocates, life coaches, celebrities and influencers, educators, journalists, and more. They may be used by mental health professionals as part of their therapeutic services, providing a resource to clients and patients to support their recovery [13]. Many prominent mental health organisations have released their own podcasts, for example, *Not Alone* by Beyond Blue (Australia), *The Mind Podcast* by Mind (UK), and *In the Open* by Mental Health America (USA).

Recent research has begun to map how health-related podcasts are being used and evaluated, including a recent scoping review by Robins et al. that identified three interventional studies for depression and anxiety with null findings [15]. But despite the plethora of mental health-related podcasts available to the public, these have not been systematically explored in terms of evaluation approaches, or for a wider range of mental health outcomes. There is a need to understand the format and structure of podcast-based interventions, to understand how mental health professionals, advocates, and researchers have utilised this medium to communicate with audiences and evaluate their impact. There is also a need to understand such podcasts’ impact upon listeners to date.

A systematic scoping review was chosen as the approach for the current study as the area had not previously been systematically reviewed, and was likely to be highly heterogeneous in terms of types of podcasts and mental health outcomes [16,17]. Furthermore, the authors wished to understand broad characteristics, concepts, and methodology, rather than answering a specific question about efficacy. The aim of this review was to determine how podcasts are being used as a platform for mental health-related interventions (‘podcast-based interventions’). This aim was achieved.

## Methods

### Protocol and registration

A scoping review protocol was developed using a template from the Joanna Briggs Institute [18] (see S1 Protocol). The protocol was not registered as this is not a requirement for scoping reviews [18], and PROSPERO does not allow registration of scoping reviews. Reporting was checked against the Preferred Reporting Items for Systematic Reviews and Meta-analyses extension for Scoping Reviews (PRISMA-ScR) [19].

### Eligibility criteria

Eligible study types were peer-reviewed journal articles, including pre-prints; conference proceedings (abstracts, presentations and papers), and theses/dissertations. Due to resourcing, only English-language results were eligible. Primary study designs only were included, but any relevant systematic reviews located during the search were scanned for any papers that might meet inclusion criteria. For the purpose of the review, ‘podcast-based interventions’ were interventional or experimental studies, where participants listened to an audio podcast, and the study targeted one or more mental health-related outcomes. Studies were required to present pre- and post-intervention data on outcomes such as: diagnosis aligned with the Diagnostic and Statistical Manual of Mental Disorders, 5th edition [20]; cognitive, behavioural, or affective symptoms of mental ill-health or distress; management of mental health issues such as self-reported access to treatments or usage of coping skills; or mental health literacy, stigma, prejudice, or discrimination. Full operationalisation is presented in S1 Protocol. Studies could involve participants of any age, sex and ethnicity, in any setting and geographical location.

### Information sources

The following databases were searched from oldest record to November 1, 2021: CENTRAL (Cochrane Central Register of Controlled Trials), EMBASE, PsycINFO, Communication and Mass Media Complete, Web of Science (all databases), and ProQuest Dissertations & Theses Global. The search was conducted again on 4 August 2024 to identify newly published records.

### Search

The search strategy was limited to abstract, title and keywords only where possible. Searches involved a combination of podcast-related search terms and mental health outcome-related terms. Example search terms from one database, PsycINFO, are presented below; all search terms are presented in S1 Protocol. Notably, the search terms included terms usually applied to podcasts with video components, such as ‘vidcast’ and ‘vodcast’. This decision was made because many overlapping terms have been used to describe podcasts [5], and there was a risk that some relevant studies might be missed if search terms were limited. Notably, video-based content was excluded as it was out of scope; such content is likely to influence audiences in a different manner to audio-only content [21,22].

(“podcast*” OR “webcast*” OR “vodcast*” OR “vidcast*” OR “mobcast*” OR “digital audio” OR “internet audio” OR “digital radio” OR “internet radio”).tw. AND (“mental health*” OR “mental illness*” OR “mental disorder*” OR schizophreni* OR psychosi* OR bipolar OR depressi* OR dysthymi* OR anxi* OR panic OR phobi* OR “obsessive compulsive” OR OCD OR trauma* OR PTSD OR disassociati* OR somati* OR “eating disorder*” OR anorex* OR bulimi* OR “binge eating” OR “personality disorder*” OR “coping skill*” OR mindful* OR psychotherapy OR therap* OR “help-seeking” OR “mental health literacy” OR psychoeducat* OR “mental health knowledge” OR stress* OR distress* OR stigma* OR prejudic* OR discrim*).tw.

### Selection of sources of evidence

S2 Table overviews the screening process. All identified records were collated and uploaded into the screening tool Covidence. Duplicates were removed. First, titles and abstracts were screened by two independent reviewers (either EC, BH or AW) for assessment against the inclusion criteria, who then reviewed full texts. Any conflicts were resolved by consensus.

If multiple reports of a same study were identified, these were grouped as one study and the report with the most complete data was classified as the primary publication. If a relevant clinical trial registration, conference abstract, or dissertation was identified, the reviewers searched for any peer-reviewed publications that may have been missed in the search. For RCTs, the reviewers manually searched for any accompanying trial registrations. Authors were contacted to request further records if necessary. Reference lists of reports that met inclusion criteria were also searched and screened.

### Data charting process

Reports meeting inclusion criteria were allocated to either EC or BH for data extraction. The reviewers used a data extraction form created in Covidence, which was pilot tested by the independent reviewers using a randomly selected article from the full text list. After data extraction, each form was checked for accuracy by another reviewer (either EC, BH, or AW) prior to marking the extraction as complete. Data were then exported to Microsoft Excel (see S2 Table) to generate descriptive statistics including frequencies and measures of central tendency.

### Data items

The data items extracted included publication type, study objectives, context of study, study design, description of podcast content and intervention, participants and recruitment procedures, definition of ‘podcast’ provided by authors, completion and attrition rate, outcomes measured, results, key conclusions drawn by authors, and limitations of the study. A full list, with definitions, is provided in S1 Protocol.

### Critical appraisal

As standard for systematic scoping reviews [18], and due to the broad range of outcomes eligible for inclusion in the review, the authors did not conduct a quality appraisal.

### Synthesis of results

Aligned with the JBI manual [18], results are presented descriptively, grouped by 1) podcast intervention features, 2) intervention methodology, and 3) key findings. Key findings are grouped broadly by intervention type.

## Results

### Selection of sources of evidence

**Fig 1:**
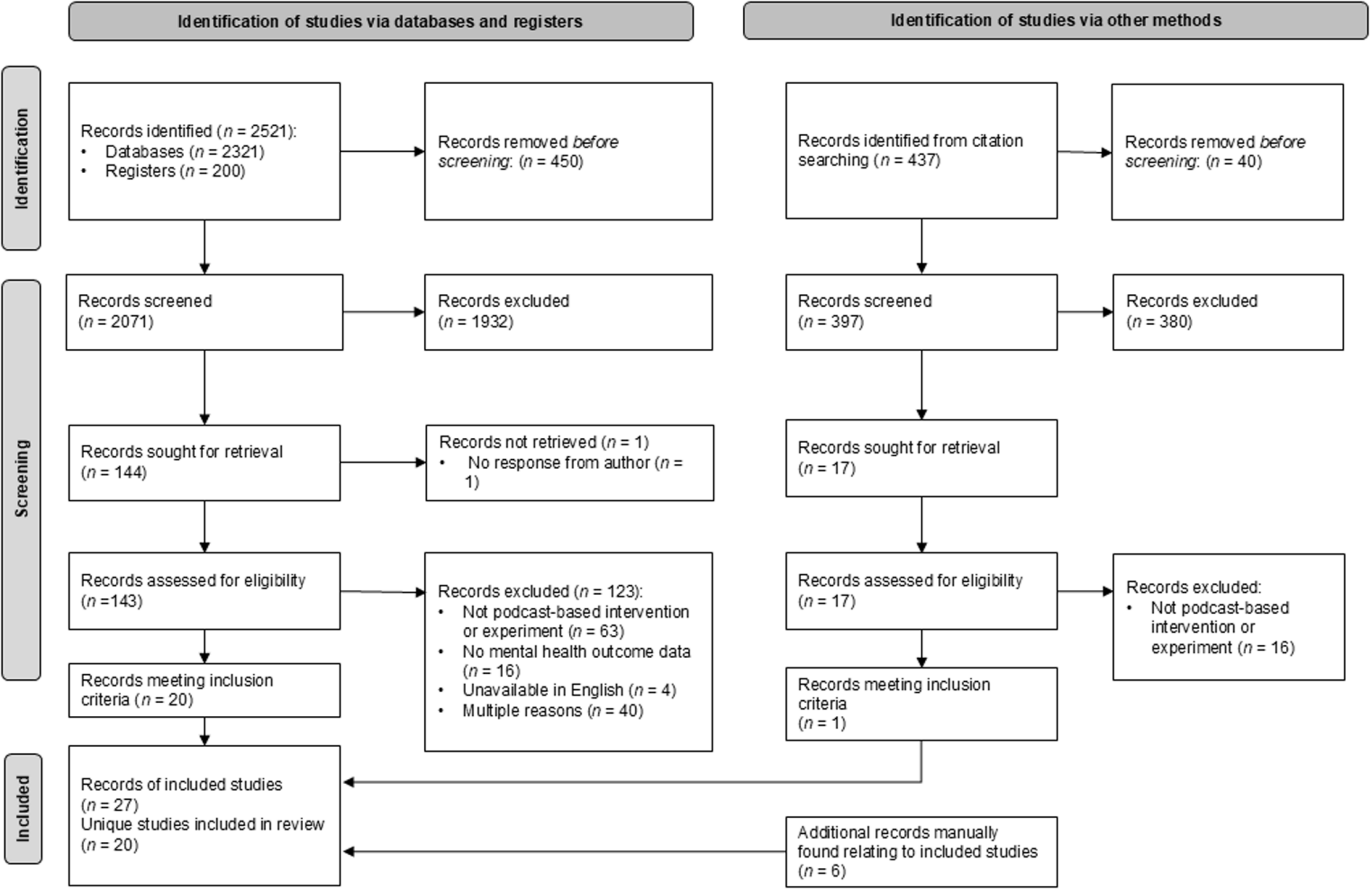
PRISMA flowchart.

Please see Fig 1 for the PRISMA flowchart. Across all searches, there were 2958 records identified, with 2468 screened after removal of duplicates.

The initial database and trial register search produced 1824 records, with 1717 records from databases, and 107 from trial registers. After removal of duplicates, 1510 titles and abstracts were screened, with 13 meeting inclusion criteria after full text review. Updated searches (searching between 1 November 2021 to 4 August 2024) identified a further 697 records, with 604 from databases and 93 from trial registers. After removal of duplicates, 566 unique titles and abstracts were screened, with seven new records meeting inclusion criteria after full text review.

An additional citation list search identified 437 citations, with 397 titles and abstracts screened after duplicates removal. One additional record met inclusion criteria after full text review.

Additional searches linked six further records from the unique studies (e.g., peer-reviewed publications published after trial registrations). After accounting for multiple reports and merging relevant records, in total, there were 20 unique studies included in the scoping review, with 27 associated records.

### Characteristics of sources of evidence

Publication characteristics are presented in Table 1. Publication occurred between 2011 and 2024, with over half (11/20, 55%) published in the last five years. Study location was most commonly the USA (10/20, 50%) and Australia (5/20, 25%). Across the 27 records, there were 13 peer reviewed-journal articles, eight dissertations, five clinical trial registrations, and one conference abstract. Eleven studies (55%) were randomised controlled trials, with the remaining nine being pre-post studies including experimental studies or non-randomised controlled trials.

**Table 1:** Characteristics of included studies.

Author and date^a^	Records	Country	Study design	Number of groups	Population	*N*	Podcast content type	Mental health outcomes
Albertson 2013	2	United States	RCT	2	Women with body image concerns	228	Meditation/mindfulness	Body image; self-compassion
Basso 2019	1	United States	RCT	2	General community	42	Meditation/mindfulness	Mindfulness; self-esteem; sleep quality; rumination; perceived stress; anxiety, depression
Bui 2023	3	United States	Pre-post study	1	Military caregivers	55	Psychoeducational/ therapeutic	Stress; depression; anxiety participation in social roles
Carrotte 2024	1	Australia	RCT	2	Students	163	Lived experience non-fiction storytelling	Stigmatising attitudes and discriminatory intentions relating to complex mental illness
Davies 2018	2	Australia	RCT	3	Students + general community	93	Meditation/mindfulness	Mindfulness; depression; anxiety; stress
Desai	1	United States	RCT	2	Healthcare providers	32	Meditation/mindfulness	Compassion satisfaction, burnout, secondary traumatic stress
Deotto 2023^b^	1	Canada	Pre-post study	1	Parents of children with neurological or neurodevelopmental disorders	13	Psychoeducational/ therapeutic	Child behavioural problems (frequency and severity)
De Wet 2020	2	Australia	RCT	2	Women (students + general community)	70	Meditation/mindfulness	Body image; self-compassion
Dunston 2021	1	United States	Pre-post study	1	Students (Nursing graduate)	30	Psychoeducational/ therapeutic	Anxiety; stress; depression; usage of wellbeing app, mental health service utilisation
Fernandez 2011	1	United States	Pre-post study	1	Students (International freshmen)	53	Educational (other)	Classroom anxiety
French 2011	1	UK	Pre-post study	1	General community	145	Psychoeducational/ therapeutic	Stigmatising beliefs about psychosis; metacognitive beliefs about paranoia; knowledge around psychosis
Kissell 2022	1	United States	Pre-post study	1	Students (pharmacists)	121	Psychoeducational/ therapeutic	Stigmatising attitudes relating to opioid use disorder
Kazan 2018	2	Australia	RCT	2	Adults who have experienced relationship separation	124	Psychoeducational/ therapeutic	Suicidal ideation, benefit finding, depression, psychological distress, help seeking attitudes, adjustment to relationship separation
Morawska 2014	1	Australia	RCT	2	Parents of children with behavioural or emotional concerns	139	Psychoeducational/ therapeutic	Perception of child’s behaviour and emotional adjustment; parenting style, parenting self-efficacy
Pownell 2014	1	UK	Pre-post study	1	General community	162	Psychoeducational/ therapeutic	Body image; perfectionism; social anxiety; self-esteem; depression; alcohol and drug use
Seiter 2021	1	United States	RCT	4	General community (MTurk panel)	695	Educational (other)	Death anxiety; behavioural change around advance care planning
Smyth 2021^c^	1	Canada	Pre-post study	Study 1: 2 Study 2: 2	Students	Study 1: 167, Study 2: 120	Meditation/mindfulness	Goal motivation
Toole 2016	1	United States	RCT	2	Women with body image concerns	80	Meditation/mindfulness	Body image; self-compassion
Wilks 2024	1	United States	RCT	3	Young people (Prolific panel)	70	Lived experience non-fiction storytelling	Stigma, self-stigma, and discriminatory intentions towards mental illness; help-seeking attitudes
Zimmermann 2017	1	New Zealand	Pre-post study	1	Advanced cancer patients	19	Meditation/mindfulness	Acceptance and psychological flexibility; mindfulness; mindful coping; meaning in life

^a^Based on primary publication (with most complete data available at the time of the search).

^b^Participants in this study were involved in a stepped care program, with a podcast-only condition being step 1; data are only presented for those who stopped the program after step 1 (*n* = 13) and thereby provided podcast-only data.

^c^This publication presented one larger study with two sub-studies. For the purpose of this review, this is classified as one study as they contain near identical methodology. The samples are aggregated in reporting aside from relevant columns in Table 1.

### Podcast intervention features

#### Definitions.

Only five studies provided a clear definition of a podcast [23–27]; all referred to an audio recording or file, and all but one [27] referenced distribution through the Internet. Three definitions also highlighted podcasts as being on-demand [23–25]. Fernandez [23] further defined podcasts as also being distributed using RSS or Atom syndication formats. In their definition, Kissell et al. [26] specified that podcasts “frequently utilize storytelling or conversations between individuals to explore a topic” (p. 2), while Pownell’s [25] definition also mentioned that podcasts are “short” and “inexpensive to produce” (p. 46).

#### Episode descriptions.

Key intervention characteristics are presented in Table 2. Across studies, participants were asked to listen to between 1 episode [24,25,28–31] and up to 28 episodes (30). The shortest reported episode length was 3 minutes [29], while the longest episodes reported were 50-60 minutes [32]. Typically, podcast episode length was between 10-20 minutes long.

**Table 2:** Podcast intervention characteristics.

Variable	Category	*N*	%
**Number of individual episodes (per group)**	1 episode	6	30%
	2 episodes	2	10%
	3 episodes	4	20%
	4 episodes	1	5%
	5 episodes	2	10%
	6 episodes	2	10%
	7 episodes	1	5%
	28 episodes	1	5%
	Not specified	1	5%
**Content of episodes**	Meditation/mindfulness exercises	8	40%
	Psychoeducation/therapeutic	8	40%
	Educational (other)	2	10%
	Lived experience non-fiction storytelling	2	10%
**Episode style**	Scripted or instructional	10	50%
	Conversational/interview	3	15%
	Documentary	2	10%
	Lecture	1	5%
	Mixed format	1	5%
	Not specified	3	15%
**How participants listened**	Lab setting	1	5%
	Online - link to website or streaming platform	8	40%
	Online - embedded in survey	6	30%
	Online – not specified	2	10%
	Multiple methods	2	10%
	Not specified	1	5%

Content of podcast intervention episodes varied significantly, with the majority seemingly being scripted or instructive; these were usually meditation or mindfulness-based podcasts. Only three [25,28,33] were explicitly described as being conversational in nature, or interview-based; two were documentary-style including clips from people with lived experience of mental health issues interspersed with narration or other audio elements [30,34]. One described a mixture of audio clips including interviews from people with lived experience and health professionals [26]. Though not always specified, episodes were described as typically involving 1-2 speakers (such as a single host guiding the listener through a mindfulness exercise). An exception was Kissel et al. [26], which described episodes as having a range of clips from different guests with various backgrounds.

As seen in Table 2, eight podcasts (40%) involved predominantly meditation or mindfulness exercises. These included guided body scans, guided imagery, relaxation and breathing exercises, attention training, and self-compassion meditations. Eight (40%) podcasts were classified as predominantly psychoeducational or therapeutic in nature. These podcasts included body image discussions based in cognitive behavioural therapy principles [25], coping skills and self-care [27,35]; psychoeducation about stress management [35,36]; brief interpersonal psychotherapy-based content around adjusting to a relationship separation [27]; parenting strategies for preventing and managing behavioural or emotional concerns in children [31,33]; and discussions about specific mental health conditions: psychosis [24] and opioid use disorder [26]. Two were focussed on storytelling with people from lived experience of mental health issues, with one person’s story presented per episode [30,34]. Other educational podcasts included audio-recorded university lectures, a.k.a. ‘coursecasting’ [23] while another involved short audio clips relating to advance care planning in the event of death [29]; both were eligible for the present review as they reported mental health-related outcomes as operationalised in S1 Protocol.

#### Comparison conditions.

As seen in Table 1, eight of the 20 studies (40%) involved only one group, while twelve studies (60%) involved at least one comparison condition. Nine (45%) had two groups, two (10%) had three groups, and one (5%) had four groups.

Of those with a control or comparison condition, half (6/12, 50%) involved some sort of comparison audio, five (5/12, 42%) involved a no-treatment or waitlist condition, and one was compared to an in-person meditation condition (the podcast was classified as the control condition [32]). Three studies (3/12, 25%) included at least one thematically different podcast as a control, externally produced and matched for episode length – including a science and philosophy podcast episode from *Radio Lab* [37], a history/pop culture podcast episode from the podcast *Stuff You Should Know* [28], and an unspecified nature podcast [30]. Others included at least one similarly themed podcast, but missing an active or key element of the intervention of interest, including different meditation exercises [38] or ‘sham’ mindfulness exercises that did not involve an attentional anchor [39], and episodes from *This American Life* and *ABC Radio National* about mindfulness and society [39]. Another involved a general interest psychology podcast created by the same institution but without an explicit focus on stigma or discrimination as seen in the intervention condition [34].

Meanwhile, two studies tested variations on the intervention itself. First, Wilks [30] compared three groups: one group were randomised to listen to any episode from a 9-part series, the second group were allowed to choose their own episode, and the third listened to a nature podcast. Seiter [29] compared different variations of similar content, testing variations on profanity, humour, and gender of the narrator.

### Intervention methodology

#### Population, sample size and attrition.

There were 2621 participants across 20 studies. Sample size ranged from 13-695 participants (median = 93, *IQR* = 53-145). Attrition ranged from 4-84%, with a median attrition rate of 42% (*IQR =* 10-50%). Both the largest sample and smallest attrition rate were from Seiter [29], who recruited on Amazon MTurk, though notably this study also had the lowest participant burden due to the shortest intervention length of only a single 3-5 minute audio clip. The highest attrition rate (84%) was reported by Kazan [27], a three-week therapeutic intervention targeting depression and suicidal ideation in people who had experienced a relationship separation; this was one of the more intensive studies, involving a three-week intervention period with six 8-13 minute episodes, and a subsequent three-week follow-up period.

Almost all studies involved convenience samples, and all but one used entirely adult samples (de Wet [38] recruited from a minimum age of 17). Samples were highly varied. Most common samples were tertiary students (5/20, 25%) and general community samples (4/20, 20%), parents or carers (3/20, 15%), and healthcare providers or trainees (3/20, 15%). Two studies (13%) specifically sampled women with body image concerns [40,41], while one study each sampled adult advanced care cancer patients [42] and people who have experienced a relationship separation [27].

Most recruited from online advertising (e.g., social media, email newsletters) and/or through tertiary student research participation pools. Only one study recruited from a clinical mental health setting, as some of Deotto et al.’s [31] participants were parents of pediatric patients from a children’s hospital; Zimmerman et al. [42] also recruited from a hospital setting, with cancer patients drawn from a public hospital oncology department. Studies typically did not provide information about participants’ mental health-related diagnoses, though in some studies, diagnosed mental health conditions were exclusion criteria [27,37]. Eight studies (40%) involved no participant payments. Five (5/20, 25%) involved monetary or gift voucher payment, whereas others involved course credits, prize draws, or a combination of these.

#### Other intervention characteristics.

Participants in most studies (16/20, 80%) listened to podcasts online. In eight studies (40%), participants listened to podcast episodes provided via a link to a website where the audio was hosted, such as a dedicated podcast distribution platform (e.g., Apple Podcasts, Soundcloud or Anchor) or another website (e.g., a university website). Six studies (6/20, 30%) involved podcast audio being embedded in a survey platform (e.g., SurveyMonkey or MTurk). Two (10%) noted participants accessed the episodes online, but did not specify how exactly they accessed the podcast.

One study was lab-based, with participants listening in-person [28]. Two studies (2/20, 10%) involved multiple modes of delivery, including initially in a lab setting with at-home practice via embedding in a survey [39], or an initial home visit plus choice of streaming or accessing the file via CD or USB [42].

Interventions were typically 1-4 weeks in length, and ranged from 3-5 minutes (a single podcast episode, excluding time taken to complete pre- and post-measures embedded in the survey [29]) to 12 weeks (with three episodes made available at the start, middle, and end of the period [35]). Only five studies (5/20, 25%) reported follow-up periods, which were 4 weeks [28,34], 3 months [27,40], and up to 6 months [33].

Most studies (15/20, 75%) did not mention any attention or manipulation checks. Three (15%) included an attention check [29,34,38], one included a manipulation check [39], while one included both [28]. Examples of attention checks included asking participants if they did anything else while listening to recordings, recall items, and directed-choice questions. Manipulation checks were only present in meditation and mindfulness studies, and included assessing perceived state mindfulness [28] or belief they had practiced mindfulness [39].

### Outcomes

#### Mental health outcomes measured.

Studies targeted a range of mental health-related outcomes (see Table 1). They heavily relied on self-report questionnaires, and no studies used diagnostic measures. Smyth et al. [28] was the only study with an objectively measured outcome, in this instance, a behavioural response to an acute stressor via the Trier Social Stress Test.

Most studies aimed to improve wellbeing and/or reduce symptoms associated with mental ill-health. The most common outcome measured was anxiety, seen in 7/20 (35%) studies, including general symptoms of anxiety, social anxiety, death anxiety and classroom-related anxiety. Other common outcomes were depressive symptoms (6/20, 30%), stress or psychological distress (5/20, 25%), body image-related variables (4/20, 20%).

Four studies (4/20, 20%) included a stigma-related outcome; two specifically measured the podcast impact on stigma towards people living with mental health issues in general, with a focus on young people [30] and those with complex mental health issues [34]. Two targeted stigma associated with a specific mental health condition: French et al. [24] explored the impact on knowledge and beliefs towards people living with psychosis, whilst Kissell et al. [26] explored attitudes towards people living with opioid use disorder.

#### Impact on mental health outcomes.

Fourteen of the 20 studies (70%) reported a statistically significant result on at least one mental health-related outcome. Findings are summarised below, by content of podcast intervention.

Seven of the eight (88%) meditation or mindfulness studies found some evidence of a positive impact of the podcast on a mental health outcome. Three of these podcasts [38,40,41] involved RCTs with guided self-compassion meditations to improve body image in adult women. The earliest of these studies, Albertson et al. [40], found that after listening to daily 20-minute meditations, women with negative body image in the intervention group experienced a significant improvement in self-compassion (large effect), body appreciation, body satisfaction, and body shame (medium effects), and appearance-contingent self-worth (small effect), compared to a waitlist control. Findings were maintained at a three-month follow-up. Later studies expanded on these findings, finding similar positive impacts of a briefer, one-week intervention length [38,41] and using a sample of women who did not necessarily have pre-existing body image concerns [38].

Other meditation and mindfulness-based podcast interventions were varied in foci but generally resulted in positive findings. Basso et al. [37] and Smyth et al. [28] explored the impact of short meditation or mindfulness podcasts, finding intervention groups had a significant decrease in mood disturbance and reduction in a behavioural stress response (but not anxiety) over time [37] and improvements in state mindfulness and goal motivation [28], compared to control conditions. Zimmermann et al. [42] studied the impact of four episodes of a meditation podcast on adults with advanced cancer, finding significant, moderate-large improvements in several mindfulness-related variables (acceptance and psychological flexibility, awareness, constructive self-distraction, and presence), and a small improvement in search of meaning, from pre-to-post intervention. To contrast, Davies et al. [39] compared six episodes of a mindfulness condition, a ‘sham’ mindfulness condition and a control podcast, finding those that listened to the mindfulness audio experienced a significant increase in only one facet of mindfulness (nonreactivity) but not others, and no significant group differences between mindfulness and sham mindfulness on any of the five facets. There were also no group differences on measures of depression, stress, or anxiety. Finally, in the study by Desai et al. [32], healthcare providers listened to five 50-60 minute episodes involving a gratitude practice; participants did not report significant improvements in compassion satisfaction, burnout, or secondary trauma stress. Comparatively, those allocated to a heartfulness meditation condition did report statistically significant improvements for burnout, secondary trauma stress, and work engagement.

Of the eight psychoeducational podcast studies, six were aimed at improving the wellbeing of listeners, whereas two were focussed on stigma-related outcomes (described below). Most studies generally had positive findings, including small-medium improvements in stress among military caregivers [36], and improvements in weight concerns and self-esteem (large effect), mood (medium effect), and social anxiety (small effect) among women [25]. Two focussed on parents: one reported medium-large improvements in child behavioural problems, parental self-efficacy and confidence [33]. The other reported no significant improvements in child behavioural problems or intensity in the podcast-only condition (step 1 or a stepped-care model), though those who completed latter, more intensive stages beyond the podcast-only condition reported significant improvements [31]. Another studied graduate nursing students and found no significant impact of listening to the podcast on anxiety, depression or stress, though all students who remained in the study were using a wellbeing app by the end of the study [35]. One study of adjustment to relationship separation [27] found no significant condition effects of the podcast versus a waitlist control on various outcomes including suicidal ideation, depression, and help-seeking attitudes, though this study was notably limited by high attrition (84%).

Two psychoeducational podcast studies and two lived experience podcast studies focussed on stigma-related outcomes. All studies reported statistically significant outcomes. Two focussed on stigma associated with a specific mental health condition via pre-post studies: French [24] reported that participants who listened to one podcast episode with psychoeducation content around psychosis reported a significant reduction in negative beliefs about hearing voices and paranoia, an increase in normalising beliefs about paranoia, and improvements in knowledge around the prevalence of psychosis, whilst Kissell et al. [26] found that student pharmacists who listened to five episodes experienced significant, small-medium improvement in attitudes towards people with opioid use disorder. However, this study did not measure stigma on a validated scale. Meanwhile, the lived experience-focussed podcasts assessed stigma and discrimination towards people with lived experience of mental health issues generally. Carrotte et al. [34] found that psychology students who listened to three episodes had significant short-term improvements in prejudice, but not tolerance and support or discriminatory intentions. Wilks [30] found that young people who listened to a single podcast episode (whether they chose the episode or not) reported small-medium significant improvements in beneficial attitudes and pessimistic attitudes; public stigma, perceived difference; and self-stigma. This study also explored whether participant choice of episodes made a difference; those given option to choose the episode reported an large improvement in a beneficial attitudes subscale, but there were no other group differences.

The remaining two podcasts had unique foci. First, Fernandez [23] conducted a naturalistic study of universities that were already using coursecasting, finding no statistically significant changes over time on classroom anxiety for participating international students. Second, Seiter [29] compared different podcast conditions around death advance care planning, with different tones and uses of humour and profanity. Each of four groups were exposed to one different, short podcast clip; there were no significant differences between any of the groups and the control condition on death anxiety or communication apprehension about death.

## Discussion

Across 20 studies, this systematic scoping review highlighted the breadth of podcast-related interventions targeting mental health-related outcomes in listeners, with most being published in the last five years. The review identified a variety of podcast types which have been studied experimentally, including meditation and mindfulness, psychoeducational content, other educational content, and lived experience non-fiction storytelling. These podcasts generally had positive impacts on a range of outcomes including increasing state mindfulness, self-compassion, stress, parenting-related and body image-related variables, and reducing stigma towards people living with mental illness. However, the relatively small number of podcast-based interventions identified in the search was somewhat surprising, considering podcasts have been produced for over 20 years at the time of writing [6]. The thousands of mental health-themed podcasts available to the public – including by well-known mental health organisations – far outnumber podcasts with published impact data. It is unlikely that this sample accurately represents mental health podcasts widely, and perhaps reflects a lack of interest in empirically measuring the impact of podcasts to date. However, as podcasts usually involve a passive, one-way listening experience – especially those designed to be universal interventions, delivered through mass media – not all mental health-themed podcasts are conceptualised as interventions with measurable mental health-related outcomes. Hence, in these instances, rigorous evaluation would not necessarily be plausible or warranted.

The podcasts identified in this review were heterogeneous, including diverse content aiming to affect a range of mostly self-reported mental health-related outcomes. Interestingly, nearly all podcast content was described or implied to be scripted – particularly those which involved meditation or mindfulness-based activities. This is despite podcasts being amenable to a range of formats, including semi-scripted or unscripted interviews and conversations, panel discussions, documentary-style storytelling, and even fictional narratives. In general, there was minimal description provided about the podcast content, limiting replicability. Few studies provided detailed descriptions about episode formats, production processes, or the usage of music, sound effects, or other elements that may contribute to a podcast’s tone or the listeners’ emotional experience or make content engaging [43]. There is a need to further explore which formats, guests, and creative elements might make podcast-based interventions engaging and effective for their intended listeners and outcomes. This might first require formative research with target audience members (e.g., co-design [44]). Such studies can help identify the ‘active’ elements of a podcast that can lead to the most change, prior to testing the podcast’s efficacy. Future mental health podcast creators and evaluators may benefit from drawing from communication and marketing literature [e.g., 45] to maximise podcast reach and impact in real-world settings, including when targeting broad populations or more selective groups [11].

This study also raises a question: what exactly can be labelled as a podcast? Most studies only involved exposure to 1-3 episodes, and the shortest podcast consisted of only a single episode of 3-5 minutes [29], which participants listened to once. This, alongside only five studies providing a clear definition of a ‘podcast’, illustrates significant conceptual overlap between podcasts and general audio-based stimuli used in interventions, and conceptual issues seen more broadly [5]. Single exposures to short clips are limited: they cannot build on messaging over time, or reinforce messages; nor can they be expected to foster a sense of connection between listeners, hosts and guests. Indeed, as podcasts typically use an RSS feed, this implies podcasts inherently have a serial nature, with multiple episodes [5,6]. Contrasting this, most studies included podcast episodes that were under 20 minutes in length [26]; this is interesting, considering the average podcast length range is between 30 and 42 minutes [46]. This likely reflects researchers’ attempts to minimise listening burden and attrition, though it may also be appropriate for their content (for example, meditation podcasts may require a shorter length). However, this does mean the present review cannot provide information about longer-form episodes and impact of listening to series over time. Ultimately, the ‘dosage’ required to make a meaningful impact on listeners is unknown.

Notably, nearly half of the studies involved meditation and mindfulness-based content, if they were described or contextualised as podcasts. This reflects a broader trend, as thousands of meditation podcasts are available, and they are popular among listeners [14]. Guided mindfulness naturally lends itself to the podcast format: these podcasts allow listeners to access a range of guided exercises, on demand, for free or at low cost. Of note, there is already a large body of research involving digitally guided meditation, including audio recordings, meditation apps, and virtual reality [47,48] that are not necessarily conceptualised as podcasts and therefore were not eligible for the current review. The positive impacts of meditation and mindfulness on mental health outcomes are well-established (e.g., [48–50], and the podcast-based studies included in this review generally also had positive findings.

Psychoeducational content was also popular, with 40% of the podcasts in the study being categorised as such. Interestingly, almost no studies used clinical samples, such as participants who have been diagnosed with a mental health condition and were seeking psychological support. This is despite podcasts increasingly being used as an adjunct to direct counselling or psychological support [13] or as part of multi-component mental health interventions [51–53]. The use of standalone or stepped care podcast-based self-help interventions is worthy of study, considering the potential to reach a wide audience at low cost. For example, Kazan [27] adapted brief interpersonal psychotherapy to the podcast format, whilst the podcast evaluated by Dunston [35] utilised cognitive behavioural therapy and mindfulness techniques; however, both studies had high attrition and neither had statistically significant outcomes. Whilst evidence-based psychological interventions can be adapted to this format, more research is needed to explore how these can be adapted effectively. As seen with smartphone apps, it is unlikely that listeners will use such podcasts to replace traditional psychological supports, as listeners may prefer individualised, tailored support rather than generic or transdiagnostic therapeutic advice [3].

Only four studies to date have explored podcasts for stigma reduction – exploring the impact of podcasts on stigmatising attitudes towards people living with psychosis [24], opioid use disorders [26] or people with lived experience of mental health issues in general [30,34]. These podcasts used both educational content and lived experience non-fiction storytelling, involving audio directly from people with lived experience. This highlights how podcasts can involve educational and contact-based stigma reduction messaging [54], which are known to be evidence-based stigma reduction strategies [55]. Podcasts can also be used for improving mental health literacy for stigma reduction purposes [34,44]. Indeed, all studies in the present review had promising findings, reporting improvements in stigmatising attitudes among listeners. However, only two of the four studies involved RCTs [30,34] and one of the four studies did not use any validated outcome measures [26]. However, as highlighted by Carrotte et al. [34,44], podcasts are a type of ‘opt-in’ media; more work is needed to understand how podcasts can maximise listener engagement, and attract listeners outside of closed research studies who hold stigmatising attitudes and may not be interested in the subject matter.

Attrition was high across the studies, with a median rate of 42%, and up to 84%. Attrition is a common issue in digital interventions [56] and raises questions about how to incentivise participants to complete such studies. Reimbursement (seen in 60% of studies) is one option, as is reducing the length or complexity of the intervention. In real world settings, many listeners will have low interest in engaging with a podcast unless it is entertaining, engaging, and relevant to their interests [10]. Consequently, listeners may not be interested in podcasts which explicitly aim to improve their wellbeing unless they are purposively help-seeking [43]. This review did not systematically extract feedback data, but as an example of uptake issues, in one study only 39% of participants reported that they would have been willing to practise the podcast-based meditation for another two weeks [41]. Indeed, some studies acknowledged target populations being time-poor [e.g., healthcare providers, 32]. Another issue is how to balance compliance with more pragmatic considerations. Most people listen to podcasts while multi-tasking, and the ability to multi-task while listening is a key feature of podcasts and a function that appeals to many listeners [10]. A laboratory setting (such as that used by Smyth et al. [28]) might increase compliance and attention, but not represent the real-life listening experience. Most studies involved participants listening online via the Internet, and usage of attention checks may help indicate how much attention participants paid to each episode. Embedding episodes in survey software can also help researchers collect data, such as time spent on a webpage, to help understand compliance.

## Limitations

The authors acknowledge the limitations of this review. As a systematic scoping review, this study was purposefully broad, and unable to make claims about the systematic evidence for and against podcast-based interventions with mental health-related outcomes, or the quality of the evidence. Rather, it assessed the current state of both published research and grey literature, the value of this area of study, and explored methodological considerations for future research. The scope was also limited to English-language articles. Data relating to safety, risk of harm, or adverse events were not extracted; this may be of interest to future researchers. Furthermore, the authors had to carefully consider how ‘podcast’ was defined in the scoping review protocol (see S1 Protocol), considering the risk of conceptual overlap [5]. Hence, it was unable to assess evidence for podcasts with video content (i.e., webcasts, vodcasts) or those involved in multi-component or hybrid interventions (e.g., a podcast provided as a learning tool in addition to an in-person intervention). Though these are likely to have benefits, it would have been difficult to compare these with standalone, audio-only podcasts.

## Conclusions

This systematic scoping review highlights how podcasts can be an effective medium for improving listeners’ wellbeing and attitudes across many types of mental health outcomes. However, podcast-based interventions were highly diverse with significant attrition. Future podcast-based intervention studies need to carefully consider the dosage, content, and pragmatic factors in order to evaluate their efficacy and ensure they are safe and appropriate for both selective and universal intervention efforts. This requires careful consideration of health communication literature to inform a theory of change relevant to the medium as well as the message [34,43]. Evaluation studies could also consider using objective or behavioural outcomes, and increasing the usage of control and comparison conditions, to improve study quality.

Future systematic reviews and meta-analyses should assess the quality of the emerging evidence base, and explore the features of podcasts which enhance the listener’s experience and engagement with the content, such as length, number of episodes, and types of content. They may also consider collating listener feedback and other acceptability data.

## Supporting information

S1 Protocol Systematic scoping review protocol. (DOCX)

S1 Table Screening flow. (XLSX)

S2 Table Extraction table. (XLSM)
