# Supplementary material for: The impact of podcast-based interventions on mental health: A systematic scoping review
Source: PLOS Ment Health. 2025 Mar 19;2(3):e0000272. doi: 10.1371/journal.pmen.0000272 (PMC12798468; doi:10.1371/journal.pmen.0000272)
Supplement: S1 Protocol — (DOCX) [file pmen.0000272.s001.docx]

Protocol

Podcast-based mental health interventions: a systematic scoping review

# Date

# October 27, 2021 (updated 8 April 2022)

# Authors

# Ms Elise Carrotte^1,2^

# Ms Beth Hobern^1,2^

# Dr Christopher Groot^2^

# Dr Fincina Hopgood^2,3^

# Dr Michelle Blanchard^2^

# Prof Lisa Phillips^2^

# ^1^Anne Deveson Research Centre, SANE, Carlton, Australia

# ^2^Melbourne School of Psychological Sciences, University of Melbourne, Parkville, Australia

# ^3^School of Humanities, Arts and Social Sciences, University of New England, Armidale, Australia

# Protocol Abstract

**Objective:** The objective of this scoping review is to understand how podcasts are being used as a platform for mental health-related interventions (‘podcast-based interventions’).

**Introduction:** A podcast is an Internet-based audio file which can be streamed or downloaded to a computer or mobile device, typically available as a series of episodes. There is a need to explore and summarise existing research in this area to inform the development of new podcast-based interventions, and how they may be evaluated.

**Inclusion criteria:** Studies will be included in the scoping review if they include an audio-only podcast-based intervention focussing on a quantitative mental health-related outcome, including symptoms, treatment or management, of mental health issues, mental health literacy or knowledge, and mental illness stigma, prejudice or discrimination. Studies will be included for any population and in any setting. Only experimental and quasi-experimental studies will be eligible.

**Methods:** Six databases will be searched: CENTRAL, EMBASE, PsycINFO, Communication and Mass Media Complete, Web of Science, and ProQuest Dissertations & Theses Global. Journal articles, conference proceedings, and dissertations will be eligible for inclusion. Databases will initially be searched for English-language results up to November 1, 2021, using a combination of search terms for podcasts and mental health-related interventions. The search will be repeated if more than 12 months pass between the search and submission for publication. Results will be screened by two independent reviewers in a two step process (title and abstract review, followed by full text review). Data extraction will include key definitions, participants, methodology, attrition rates, and results.

# Introduction

A podcast is an Internet-based audio file which can be streamed or downloaded to a computer or mobile device, typically available as a series of episodes. The word ‘podcast’ is a portmanteau of the word ‘iPod’ and ‘broadcast’. Podcasts are generally publicly distributed through an ‘RSS Feed’ which allow individual audio files to be broadcast to different distribution apps, such as Apple Podcasts and Spotify. They may also be downloaded as individual audio files from sources such as a podcast program’s website.

Podcasts were first introduced in the early 2000s, and have shown significant popularity in the last few years. As of 2021, there an estimated two million podcasts series and over 48 million individual podcast episodes available (Winn, 2021). Furthermore, in Australia, an estimated 31% of adults have listened to a podcast within the last month, with 19% listening in the past week (Australian Broadcasting Commission, 2019).

There are many unique features of podcasts that contribute to why listeners choose to tune in (Perks et al., 2019). According to previous research, listeners find podcasts to be engaging, diverse, and customisable, with popular podcasts covering everything from true crime to celebrity gossip to meditation (Australian Broadcasting Commission, 2019; Perks et al., 2019; Perks & Turner, 2018). Podcasts are also highly accessible and portable, with many listeners choosing to multitask while listening, and some listeners choosing to stop and start episodes or even listen to material repeatedly (Cho et al., 2017; Perks & Turner, 2018).

As a flexible audio medium, podcasts allow opportunities for both educational messaging and direct emotional appeal through a variety of formats including interviews, documentary-style podcasts, and panel discussions. Podcasts have potential to be effective platforms for psychological and mental health-related interventions, including those which aim to reduce symptoms of mental health issues, increase mental health literacy or knowledge, or reduce stigmatising attitudes towards people affected by mental health issues.

There are a limited number of podcast-based interventions that have been identified in the mental health context. For example, podcast-based cognitive and behavioural interventions have been found to reduce performance anxiety when speaking a new language (Hamzaoğlu & Koçoğlu, 2016), increase physical activity (Mailey et al., 2016), and reduce stigmatising attitudes towards people experiencing psychosis (French et al., 2011). In the context of medical education, podcasts have been used to increase students’ knowledge, demonstrated through improvements in test scores (Cho et al., 2017).

There is a need to explore and summarise existing research in this area. As podcasts can be highly diverse, there is a need to understand the format and structure of podcast-based interventions, in order to understand how researchers have utilised this medium to communicate with audiences. It is unclear how researchers might define podcasts, how long their episodes or series may be, and what content has been studied. There is also a need to understand how such podcasts have been evaluated to understand their impact. These findings can inform the development of new podcast-based interventions, and how they may be evaluated.

A systematic scoping review was chosen due to the following reasons (Munn et al., 2018; Peters et al., 2015):

- To the authors’ knowledge, this body of literature has not yet been reviewed, and is likely to exhibit a heterogeneous nature.
- There is a need to identify and map available evidence in this field, to understand the scope of this body of literature broadly
- There is a need to understand characteristics and concepts, definitions, and reporting of the existing body of literature, rather than answering a precise question

A preliminary search of conducted of PsycINFO, Embase, CENTRAL, Communication and Mass Media Complete, Web of Science (all databases, including Medline), and ProQuest Dissertations and Theses Global was conducted on October 25, 2021. The search terms ‘*podcast*’* and ‘*scoping OR systematic*’, limited to titles only, were used. All searches returned zero results. This search identified no current or underway systematic or scoping reviews on the topic. Additionally, PROSPERO was searched on the same date using the term ‘podcast’. This search returned 44 protocols, but none were systematic review protocols specific to podcast-based interventions.

The objective of this scoping review is to understand the extent of the literature around podcast usage for mental health-related interventions (‘podcast-based interventions’).

# Review question

The overarching review question is: how are podcasts being used as a platform for mental health-related interventions (‘podcast-based interventions’)?

Specific sub-questions are:

1. What formats have the podcast-based interventions involved, including definitions and types of ‘podcasts’, length of episodes, number of episodes, and content of episodes?
2. For what types of mental health-related outcomes have podcast-based interventions targeted?
3. What are the attrition rates of these podcast-based interventions?
4. Is there any evidence regarding the effects of podcast-based interventions on mental health-related outcomes (specifically: symptoms of mental health issues, treatment or management of mental health issues, mental health literacy or knowledge, and/or stigma and discrimination)?

# Keywords

Podcasts; webcast; internet radio; stigma; mental illness

# Eligibility criteria

### Participants

There are no inclusion/exclusion criteria specific to participants. Participants of any age, sex and ethnicity will be eligible, in any setting. It is anticipated that most interventions will target either adolescents or adults.

### Concept

*Podcasts*

The key concept is the usage of podcasts as a platform for mental health-related interventions. As described above, a podcast is an audio file made available on the Internet for streaming or downloading to a computer or mobile device, typically available as a series of episodes. Variations include:

- 'Webcasts’, where media are streamed via the Internet, which may be live or on demand. Examples of webcasts include live-streams of lectures and events. Webcasts may contain visuals or be audio-only.
- ‘Vodcasts’, also known as ‘vidcasts’ or ‘video podcasting’, are video clips designed to be viewed via mobile device. They may also involve RSS syndication methods and are considered the video equivalent to a podcast.

Podcasts are similar to traditional radio broadcasting, which uses radio waves to transmit audio to reach an audience. Some radio programs are streamed through the Internet (‘digital radio’), or are later uploaded as podcasts for on-demand listening, and can be considered podcasts.

All variations of podcast-based interventions or experiments will be included regardless of dosage/duration/timing etc. However, for the purpose of this review, only audio-based podcast interventions will be considered. If an intervention includes an audio-only component as well as a video + audio component (i.e. a vodcast/vidcast), only the audio component will be included in the review if data are available for this component specifically.

Studies including only part of an intervention will be included if data are presented meeting the inclusion criteria. Further, podcast-based interventions do not have to be streamed via the Internet to be eligible for the review; for example, audio files may be sent to participants for direct download or participants may be exposed to them in a controlled setting.

To summarise, the inclusion criteria are:

- Interventions or experiments listening to a podcast: an audio-only file which may be made available on the Internet for streaming or downloading to a computer or mobile device, noting that
  - the researchers somewhere in the study describe or contextualise the audio file as a *podcast, vodcast, vidcast, webcast, mobcast or internet/digital radio broadcast*somewhere in the study documentation, or
  - if not explicitly called a podcast in the study - the audio file is known to be available and branded as a podcast (e.g. available for streaming on a podcasting platform), or the audio file as described very closely matches the description of known podcasts (e.g. mindfulness or sleep meditation podcasts) at the authors’ discretion
- Data are provided specific to an audio-only podcast rather than being aggregated as a multi-component intervention (e.g. a podcast + app, a podcast + therapeutic session) or with a visual component (e.g. a podcast + lecture slides, a podcast + meditative animation)

Exclusion criteria:

- Traditional radio broadcasts that do not also meet the definition of a podcast
- Webcasts, vodcasts, and other forms of video broadcasting without an audio-only podcast component
- Audio recordings are music, sound effects or soundscapes alone (e.g. intervention involves participants listening to music)
- Studies using a podcast as a control-group only, unless the control group has an ‘active’ component that would theoretically impact on the mental health outcome(s)
- The intervention or experiment simply has some kind of audio recording prompt or instruction that is not also described or contextualised as a podcast
- The intervention or experiment involves participants creating or producing a podcast, rather than listening to a podcast

*Mental health-related outcomes*

This systematic review will include any intervention studying a ‘mental health-related outcome’. For the purpose of this review, a ‘mental health-related outcome’ is defined as any domain relevant to mental health issues (including diagnosed mental disorders, mental illness, or psychological distress). These include, but are not limited to:

1. Cognitive, emotional or behavioural symptoms of mental health issues aligned with the Diagnostic and Statistical Manual of Mental Disorders, 4^th^ edition (DSM-IV) or 5^th^ edition (DSM-5)
2. Treatment or management of mental health issues
3. Mental health literacy and knowledge
4. Mental illness stigma, prejudice and discrimination

All relevant outcomes will be extracted through the review. Outcomes may align with any of the following outcome domains and may be a primary or secondary outcome:

- Diagnosis of mental disorders, aligned with the following sections of the DSM-5, or equivalent sections of the DSM-IV: schizophrenia spectrum and other psychotic disorders, bipolar and related disorders, depressive disorders, anxiety disorders, obsessive-compulsive and related disorders, trauma- and stressor-related disorders, substance-related and addictive disorders, dissociative disorders, somatic symptom and related disorders, feeding and eating disorders, personality disorders, neurodevelopmental disorders^[[1]](#footnote-1)^
- Cognitive/behavioural/affective symptoms of mental ill-health, such as:
  - self-reported or observed symptom severity (may be clinical or subclinical)
  - self-reported or observed degree of psychological distress or stress
  - self-reported or observed experience of the psychological experience of chronic pain
- Management of mental health issues, such as:
  - self-reported access to treatments for mental health issues such as psychotherapy or medication, or intention to access
  - self-reported usage of coping skills or techniques for the purpose of managing or improving mental health
  - self-reported confidence, agency, or self-efficacy in management of mental health issues, or carer burden for mental health carers
- Mental health literacy or knowledge
  - scores on questionnaires, checklists, exams or other assessments of mental health literacy or knowledge (including knowledge acquisition among psychology students or other students)
- Mental illness-related stigma, prejudice or discrimination
  - self-reported or observed degree of stigma, internalised stigma, prejudice, and actual or intended discriminatory behaviours

Exclusion criteria:

- Studies pertaining only to diagnosis or specific symptoms of the following disorders, aligned with DSM-5 chapters or equivalent DSM-IV sections: elimination disorders, sleep-wake disorders, sexual dysfunctions, gender dysphoria, disruptive, impulse-control and conduct disorders, neurocognitive disorders, paraphilic disorders, other mental disorders, medication-induced movement disorders and other adverse effects of medication, other conditions that may be a focus of clinical attention
- Studies that are specific to physical or genetic markers of mental health issues, such as weight changes or hormone levels
- Studies that focus on psychological or physical reactions that are not contextualised in terms of mental health-related outcomes defined above, such as studies of exercise for the purpose of weight loss rather than for psychological benefits, or studies of sleep that do not contextualise sleep concerns as a cause or symptom of a mental health issue defined above
- Studies that focus on broad domains such as quality of life, unless contextualised in terms of mental health-related outcomes (as defined above)
- Studies that only focus on acceptability of a podcast, or other process-based evaluation, and do not also present intervention or experimental data
- Studies that only present data on reach or engagement of a podcast (such as number of downloads)

Outcomes will be considered using any time point and using any scale. The type of measurement does not form part of the review inclusion criteria, but will be extracted. Types of main outcome measures to be extracted include:

- Statistical significance (*p* values)
- The magnitude and direction of estimates of effect (e.g. relative risk reductions, odds ratios, standardised effect sizes)
- Differences in mean and standard deviations pre- and post-study

There does not have to be a comparator for inclusion in the review. Any comparators, if present, will be eligible for inclusion in the review, including intervention vs placebo, vs control (including no intervention, wait-list control, or usual care), or versus another intervention.

### Context

The scoping review is open to any context, and will include literature:

- In any geographical location
- In any setting
- Relating to any cultural/sub-cultural, racial or gender-based factors

### Types of Sources

This scoping review will consider both experimental and quasi-experimental study designs including randomized controlled trials, non-randomized controlled trials, before and after studies and interrupted time-series studies.

Types of studies to be excluded are:

- Observational studies, including cross-sectional studies, case studies, case control studies and cohort studies.
- Literature reviews
- Qualitative studies
- Editorials or opinion pieces (unless also presenting data related to an intervention or experimental study)

# Methods

The proposed scoping review will be conducted in accordance with the JBI methodology for scoping reviews (Peters et al., 2015).

### Search strategy

The full search strategy is available in Appendix I.

The search strategy will aim to locate both published and unpublished studies. The search strategy has been developed, tested and refined with support from the University of Melbourne library services. Only English language databases will be searched, due to resourcing limitations within the team. Only English-language results will be eligible.

The following types of publications will be eligible for inclusion in the review:

- Journal articles, including pre-prints
- Conference proceedings (abstracts, presentations and papers)
- Theses and dissertations.

*Electronic searches:*

The following databases will be searched:

1. CENTRAL (Cochrane Central Register of Controlled Trials)
2. EMBASE
3. PsycINFO
4. Communication and Mass Media Complete
5. Web of Science
6. ProQuest Dissertations & Theses Global

The following restrictions will be placed on electronic searches:

- Publication date: oldest – November 1, 2021 (or, in the event of delays, oldest – date of search)

The search strategy (see Appendix I), uses a combination of terms related to podcasts and terms related to mental health-related outcomes, combined with ‘AND’. Search terms will be limited to abstract, title, and keywords only where possible. The general search terms are:

1. *Search terms related to podcasts:*

("podcast*" OR "webcast*" OR "vodcast*" OR "vidcast*" OR "mobcast*" OR "digital audio" OR "internet audio" OR "digital radio" OR "internet radio")

AND

1. *Search terms related to mental health domains:*

("mental health*" OR "mental illness*" OR "mental disorder*" OR schizophreni* OR psychosi* OR bipolar OR depressi* OR dysthymi* OR anxi* OR panic OR phobi* OR "obsessive compulsive" OR OCD OR trauma* OR PTSD OR disassociati* OR somati* OR "eating disorder*" OR anorex* OR bulimi* OR "binge eating" OR "personality disorder*" OR "coping skill*" OR mindful* OR psychotherapy OR therap* OR "help-seeking" OR "mental health literacy" OR psychoeducat* OR "mental health knowledge" OR stress* OR distress* OR stigma* OR prejudic* OR discrim*)

The search will be re-run close to publication if the initial search date is more than 12 months from submission. Any newly published articles will be reviewed according to the same process.

### Study/Source of Evidence selection

Following the search, all identified citations will be collated and uploaded into the screening tool Covidence (licensed through the University of Melbourne). Any duplicates will be removed. For study selection, the following process will be implemented:

1. Title and abstract screening

Following a pilot test, titles and abstracts will then be screened by two independent reviewers (EC and BH) for assessment against the inclusion criteria for the review.

The independent reviewers will screen each article, and mark each title and abstract as relevant or irrelevant. Articles will proceed to full text review if both reviewers mark them as relevant. They will be excluded if both mark as irrelevant. Any disagreements will be discussed and resolved by consensus, or if consensus cannot be reached, by an independent third reviewer (LP).

1. Full text review

Potentially relevant sources will be retrieved in full and their citation details imported into Covidence. The two independent reviewers (EC and BH) will read all articles that proceed to full text review and mark them as either relevant or irrelevant in Covidence. As above, articles will be removed if they are irrelevant, and LP will resolve any disagreements after discussion.

Further investigation will occur as follows:

- Systematic reviews: Systematic reviews around relevant topics will be read to identify any relevant articles included in the review. For example, if a systematic review is around app-based interventions and mentions that podcasts are included, its reference list will be scanned for any relevant articles.
- Unavailable papers, or papers missing results or information: Authors may be contacted to request further information if required, such as missing results or information about methodology. Authors will be contacted a maximum of two times via email; if they do not respond to a follow up email within a month, no further contact will be made.
- Conference proceedings and dissertations: The reviewers will check if any posters, slides, reports, or publications from the same study are published, and download these if available. Authors may be contacted as above. If no further publications are available, the abstract or dissertation may be used as the only unit of analysis and extracted.
- Clinical trial registrations: If a study is complete, the reviewers will check for any attached data or any clinical study reports, conference proceedings, reports or publications are available from the same study.  If study is not complete or no publications are yet available, the reviewers check again if more than 12 months pass between the initial search and publication. Authors may be contacted as above.

Reference lists of any journal articles included in the final systematic review will also be searched, and screened using the same systematic process in Covidence. These reference lists will downloaded and reviewed in Covidence. The reference lists of dissertations will be subject to title screening; any titles deemed relevant will be downloaded and screened systematically in Covidence.

The results of the search and the study inclusion process will be reported in full in the final scoping review and presented in a Preferred Reporting Items for Systematic Reviews and Meta-analyses extension for scoping review (PRISMA-ScR) flow diagram*.*

### Data Extraction

Articles meeting full text inclusion criteria will be allocated to either EC or BH for data extraction. Data will be extracted from articles included in the scoping review using a data extraction Microsoft Excel spreadsheet developed by the reviewers. The data extracted will include specific details about the participants, concept, context, study methods and key findings relevant to the review questions. The second reviewer will check all extracted data and any changes will be discussed before the reviewers mark extraction complete in Covidence.

A draft extraction form is provided (see Appendix II*).*The draft data extraction form will be modified and revised as necessary during the process of extracting data from each included evidence source. Modifications will be detailed in the scoping review. Any disagreements that arise between the reviewers will be resolved through discussion, or with an additional reviewer’s input (LP). If appropriate, authors of papers will be contacted to request missing or additional data, where required.

### Data Analysis and Presentation

# As podcasts are a novel medium for mental health-related interventions, it is anticipated that studies will be very heterogeneous. Data will be synthesised aligned with the scoping review’s research questions.

# This will include:

# Description of sample, including total number of studies, and key characteristics of the methodology of the studies (tabulated)

# Description of:

# Types of podcasts used, including key definitions used

# Content of podcast-based interventions

# Number of episodes and length of episodes

# Description of mental health-related outcomes that were targeted

# Description of completion and attrition rates

# Description of methodologies and effects of podcast-based interventions on mental health-related outcomes

# As outlined by the JBI manual (Peters et al., 2015), thematic analysis or synthesis is beyond the scope of scoping reviews. However, depending on the nature of the studies identified, an approach such as a descriptive qualitative content analysis, or framework synthesis, may be warranted. If this is the case, this will be outlined in updates to the protocol (see Appendix III).

# **Presentation of results**

# Data will be presented aligned with the PRISMA-ScR Checklist (Tricco et al., 2018). This will include:

# A structured summary, rationale for the scoping review, objectives, and methodology

# The PRISMA-ScR extension flowchart.

# Table of key parameters, including number of studies, types of studies, populations identified, description of podcast-based interventions, outcomes studied

# Synthesis of results

# Discussion and conclusions

# References

Australian Broadcasting Commission. (2019). *Podcast research 2019*.

Cho, D., Cosimini, M., & Espinoza, J. (2017). Podcasting in medical education: a review of the literature. *Korean Journal of Medical Education*, *29*(4), 229–239. https://doi.org/10.3946/kjme.2017.69

French, P., Hutton, P., Barratt, S., Parker, S., Byrne, R., Shryane, N., & Morrison, A. P. (2011). Provision of online normalising information to reduce stigma associated with psychosis: Can an audio podcast challenge negative appraisals of psychotic experiences? *Psychosis*, *3*(1), 52–62. https://doi.org/10.1080/17522431003717683

Hamzaoğlu, H., & Koçoğlu, Z. (2016). The application of podcasting as an instructional tool to improve Turkish EFL learners’ speaking anxiety. *Educational Media International*, *53*(4), 313–326. https://doi.org/10.1080/09523987.2016.1254889

Mailey, E. L., Huberty, J., & Irwin, B. C. (2016). Feasibility and effectiveness of a web-based physical activity intervention for working mothers. *Journal of Physical Activity and Health*, *13*(8), 822–829. https://doi.org/10.1123/jpah.2015-0643

Munn, Z., Peters, M. D. J., Stern, C., Tufanaru, C., McArthur, A., & Aromataris, E. (2018). Systematic review or scoping review? Guidance for authors when choosing between a systematic or scoping review approach. *BMC Medical Research Methodology*, *18*(1), 1–7. https://doi.org/10.1186/s12874-018-0611-x

Perks, L. G., & Turner, J. S. (2018). Podcasts and productivity: a qualitative uses and gratifications study. *Mass Communication and Society*, *22*(1), 96–116. https://doi.org/10.1080/15205436.2018.1490434

Perks, L. G., Turner, J. S., & Tollison, A. C. (2019). Podcast Uses and Gratifications Scale development. *Journal of Broadcasting & Electronic Media*, *63*(4), 617–634. https://doi.org/10.1080/08838151.2019.1688817

Peters, M. D. J., Godfrey, C. M., Khalil, H., McInerney, P., Parker, D., & Soares, C. B. (2015). Guidance for conducting systematic scoping reviews. *International Journal of Evidence-Based Healthcare*, *13*(3), 141–146. https://doi.org/10.1097/XEB.0000000000000050

Tricco, A. C., Lillie, E., Zarin, W., O’Brien, K. K., Colquhoun, H., Levac, D., Moher, D., Peters, M. D. J., Horsley, T., & Weeks, L. (2018). PRISMA extension for scoping reviews (PRISMA-ScR): checklist and explanation. *Annals of Internal Medicine*, *169*(7), 467–473.

Winn, R. (2021). *2021 Podcast stats & facts*. Podcast Insights. https://www.podcastinsights.com/podcast-statistics/

# Appendices

### Appendix I: Search strategy

**Search Strategy**

The search strategy uses a combination of terms related to podcasts and terms related to mental health outcomes, combined with ‘AND’. Subject terms will be included where relevant, and ‘exploded’ wherever appropriate.

For databases, search terms will be narrowed to title, abstract and keywords where possible, or title and abstract at a minimum. Relevant subject terms and/or keywords will also be searched. The limitations to title, abstract and keywords intends to remove a large amount of irrelevant search results identified in testing which were not about podcasts, but instead included a promotional link to the journal’s podcast or an author’s podcast.

The basic search terms are presented below:

1. *Search terms related to podcasts:*

podcast* OR webcast* OR vodcast* OR vidcast* OR mobcast* OR "digital audio" OR "internet audio" OR "digital radio" OR "internet radio"

1. *Search terms related to mental health domains:*

"mental health*" OR "mental illness*" OR "mental disorder*" OR schizophreni* OR psychosi* OR bipolar OR depressi* OR dysthymi* OR anxi* OR panic OR phobi* OR "obsessive compulsive" OR OCD OR trauma* OR PTSD OR disassociati* OR somati* OR "eating disorder*" OR anorex* OR bulimi* OR "binge eating" OR "personality disorder*" OR "coping skill*" OR mindful* OR psychotherapy OR therap* OR "help-seeking" OR "mental health literacy" OR psychoeducat* OR "mental health knowledge" OR stress* OR distress* OR stigma* OR prejudic* OR discrim*

The following databases will be searched:

1. CENTRAL (Cochrane Central Register of Controlled Trials)
2. EMBASE^[[2]](#footnote-2)^
3. PsycINFO
4. Communication and Mass Media Complete
5. Web of Science^[[3]](#footnote-3)^ (full catalogue, which includes Medline)
6. ProQuest Dissertations & Theses Global^[[4]](#footnote-4)^

The following restrictions will be placed on the initial electronic search:

- Publication date: oldest – November 1, 2021 (or, in the event of delays, oldest – date of search

Specific search strategies for each database are presented below.

| **Database** | **Podcast Terms** | **Mental Health Domains** | **Notes** |
| --- | --- | --- | --- |
| **PsycINFO** | ("podcast*" OR "webcast*" OR "vodcast*" OR "vidcast*" OR "mobcast*" OR "digital audio" OR "internet audio" OR "digital radio" OR "internet radio").tw. | ("mental health*" OR "mental illness*" OR "mental disorder*" OR schizophreni* OR psychosi* OR bipolar OR depressi* OR dysthymi* OR anxi* OR panic OR phobi* OR "obsessive compulsive" OR OCD OR trauma* OR PTSD OR disassociati* OR somati* OR "eating disorder*" OR anorex* OR bulimi* OR "binge eating" OR "personality disorder*" OR "coping skill*" OR mindful* OR psychotherapy OR therap* OR "help-seeking" OR "mental health literacy" OR psychoeducat* OR "mental health knowledge" OR stress* OR distress* OR stigma* OR prejudic* OR discrim*).tw. | Combine with AND  No specific subject term identified for podcast or webcast search terms |
| **Embase** | ("podcast*" OR "webcast*" OR "vodcast*" OR "vidcast*" OR "mobcast*" OR "digital audio" OR "internet audio" OR "digital radio" OR "internet radio").tw.  OR  Webcast/ | ("mental health*" OR "mental illness*" OR "mental disorder*" OR schizophreni* OR psychosi* OR bipolar OR depressi* OR dysthymi* OR anxi* OR panic OR phobi* OR "obsessive compulsive" OR OCD OR trauma* OR PTSD OR disassociati* OR somati* OR "eating disorder*" OR anorex* OR bulimi* OR "binge eating" OR "personality disorder*" OR "coping skill*" OR mindful* OR psychotherapy OR therap* OR "help-seeking" OR "mental health literacy" OR psychoeducat* OR "mental health knowledge" OR stress* OR distress* OR stigma* OR prejudic* OR discrim*).tw. | Combined with AND |
| **CENTRAL** | [With drop-down selection ‘Title abstract keyword]  "podcast*" OR "webcast*" OR "vodcast*" OR "vidcast*" OR "mobcast*" OR "digital audio" OR "internet audio" OR "digital radio" OR "internet radio"  OR  Webcast/ | [With drop-down selection ‘Title abstract keyword]  "mental health*" OR "mental illness*" OR "mental disorder*" OR schizophreni* OR psychosi* OR bipolar OR depressi* OR dysthymi* OR anxi* OR panic OR phobi* OR "obsessive compulsive" OR OCD OR trauma* OR PTSD OR disassociati* OR somati* OR "eating disorder*" OR anorex* OR bulimi* OR "binge eating" OR "personality disorder*" OR "coping skill*" OR mindful* OR psychotherapy OR therap* OR "help-seeking" OR "mental health literacy" OR psychoeducat* OR "mental health knowledge" OR stress* OR distress* OR stigma* OR prejudic* OR discrim* | Combined with AND  Combined search: 109 results* |
| **Communication and Mass Media Complete** | TI ("podcast*" OR "webcast*" OR "vodcast*" OR "vidcast*" OR "mobcast*" OR "digital audio" OR "internet audio" OR "digital radio" OR "internet radio")  OR  AB ("podcast*" OR "webcast*" OR "vodcast*" OR "vidcast*" OR "mobcast*" OR "digital audio" OR "internet audio" OR "digital radio" OR "internet radio")  OR  DE (PODCASTS)  OR  DE (INTERNET RADIO)  OR  DE (WEBCASTING) | TI ("mental health*" OR "mental illness*" OR "mental disorder*" OR schizophreni* OR psychosi* OR bipolar OR depressi* OR dysthymi* OR anxi* OR panic OR phobi* OR "obsessive compulsive" OR OCD OR trauma* OR PTSD OR disassociati* OR somati* OR "eating disorder*" OR anorex* OR bulimi* OR "binge eating" OR "personality disorder*" OR "coping skill*" OR mindful* OR psychotherapy OR therap* OR "help-seeking" OR "mental health literacy" OR psychoeducat* OR "mental health knowledge" OR stress* OR distress* OR stigma* OR prejudic* OR discrim*)  OR  AB ("mental health*" OR "mental illness*" OR "mental disorder*" OR schizophreni* OR psychosi* OR bipolar OR depressi* OR dysthymi* OR anxi* OR panic OR phobi* OR "obsessive compulsive" OR OCD OR trauma* OR PTSD OR disassociati* OR somati* OR "eating disorder*" OR anorex* OR bulimi* OR "binge eating" OR "personality disorder*" OR "coping skill*" OR mindful* OR psychotherapy OR therap* OR "help-seeking" OR "mental health literacy" OR psychoeducat* OR "mental health knowledge" OR stress* OR distress* OR stigma* OR prejudic* OR discrim*) | Combine with AND  This database does not have the equivalent title, abstract, keyword (.tw.) search as the Ovid databases. Separate title + abstract searches will be used in place, alongside subject headings identified in the database’s thesaurus. |
| **Web of Science** | TS=("podcast*" OR "webcast*" OR "vodcast*" OR "vidcast*" OR "mobcast*" OR "digital audio" OR "internet audio" OR "digital radio" OR "internet radio") | TS=("mental health*" OR "mental illness*" OR "mental disorder*" OR schizophreni* OR psychosi* OR bipolar OR depressi* OR dysthymi* OR anxi* OR panic OR phobi* OR "obsessive compulsive" OR OCD OR trauma* OR PTSD OR disassociati* OR somati* OR "eating disorder*" OR anorex* OR bulimi* OR "binge eating" OR "personality disorder*" OR "coping skill*" OR mindful* OR psychotherapy OR therap* OR "help-seeking" OR "mental health literacy" OR psychoeducat* OR "mental health knowledge" OR stress* OR distress* OR stigma* OR prejudic* OR discrim*) | Make sure to search in All Databases not just the WoS Core Collection  No .tw. search but WoS has option for ‘Topic’ search which includes title, abstract, author keywords, and Keywords Plus  Web of Science does not use subject terms or automatic term mapping.  Combined with AND |
| **ProQuest Dissertations and Theses Global** | ti("podcast*" OR "webcast*" OR "vodcast*" OR "vidcast*" OR "mobcast*" OR "digital audio" OR "internet audio" OR "digital radio" OR "internet radio")  OR  ab("podcast*" OR "webcast*" OR "vodcast*" OR "vidcast*" OR "mobcast*" OR "digital audio" OR "internet audio" OR "digital radio" OR "internet radio")  OR  diskw(podcast)  OR  diskw(webcasting)  OR  diskw(internet radio) | ti("mental health*" OR "mental illness*" OR "mental disorder*" OR schizophreni* OR psychosi* OR bipolar OR depressi* OR dysthymi* OR anxi* OR panic OR phobi* OR "obsessive compulsive" OR OCD OR trauma* OR PTSD OR disassociati* OR somati* OR "eating disorder*" OR anorex* OR bulimi* OR "binge eating" OR "personality disorder*" OR "coping skill*" OR mindful* OR psychotherapy OR therap* OR "help-seeking" OR "mental health literacy" OR psychoeducat* OR "mental health knowledge" OR stress* OR distress* OR stigma* OR prejudic* OR discrim*)  OR  ab("mental health*" OR "mental illness*" OR "mental disorder*" OR schizophreni* OR psychosi* OR bipolar OR depressi* OR dysthymi* OR anxi* OR panic OR phobi* OR "obsessive compulsive" OR OCD OR trauma* OR PTSD OR disassociati* OR somati* OR "eating disorder*" OR anorex* OR bulimi* OR "binge eating" OR "personality disorder*" OR "coping skill*" OR mindful* OR psychotherapy OR therap* OR "help-seeking" OR "mental health literacy" OR psychoeducat* OR "mental health knowledge" OR stress* OR distress* OR stigma* OR prejudic* OR discrim*) | This database does not have the equivalent title, abstract, keyword (.tw.) search as the Ovid databases. Separate title + abstract searches will be used in place  Combined with AND |

### Appendix II: Draft data extraction instrument

A Covidence extraction form was created with the following headings, with one study per row. This is based on the *JBI template source of evidence details, characteristics and results extraction* instrument. If there are multiple publications from the same study, the studies themselves (rather than the individual publications) will be the principal unit of interest.

Data to be extracted include the following:

- Name of data extractor and date of extraction
- Publication type (journal article, conference proceedings, dissertation)
- Objectives of study
- Citation details (e.g. author/s, date, title, journal, volume, issue, pages)
- Country where study was held
- Context
  - Year of data collection
  - Study design (e.g., pre-post study, non-randomised trial, randomised controlled trial)
  - Description of intervention groups
    - Number of intervention groups
    - Number of participants in each intervention group
    - How participants were allocated to groups
  - Recruitment procedures
  - Definition of ‘podcast’ as provided by the authors
  - Description of podcast-based intervention
    - Number and length of episodes (e.g. 3 episodes, 15 minutes each)
    - Length of intervention (e.g. six weeks)
    - Content of episodes
    - How participants listened to episodes
    - Any control/comparison group conditions
- Participants
  - mean age/age range
  - gender/sex
  - other relevant demographic information (e.g. diagnosis, ethnic background)
  - any eligibility criteria for participants
  - Completion and attrition rate
- Data/Results extracted from source of evidence
  - Statistical analyses (unit of analysis, methods used)
  - Outcomes measured
    - Name of variable
    - Description of measurement tool[s]
    - Timing of measurement)
  - Results
    - Summary data for each intervention group (e.g. statistical significance [*p* values], effect sizes, differences in means)
  - Key conclusions drawn by authors
- Any correspondence required (e.g. request further data)

### Appendix III: Summary of updates to Protocol

The following updates were made to the initial Protocol during the initial screening and extraction process, and were discussed and approved by the study team.

| **Section** | **Change** |
| --- | --- |
| Inclusion and exclusion criteria | The definition of ‘podcast’ as part of inclusion/exclusion criteria was revised and updated due to challenges differentiating between podcasts and audio-based interventions during screening.  The following text was added to inclusion criteria:   - the researchers somewhere in the study describe or contextualise the audio file as a *podcast, vodcast, vidcast, webcast, mobcast or internet/digital radio broadcast*somewhere in the study documentation, or - if not explicitly called a podcast in the study – the audio file is known to be available and branded as a podcast (e.g. available for streaming on a podcasting platform), or the audio file as described very closely matches the description of known podcasts (e.g. mindfulness or sleep meditation podcasts) at the authors’ discretion - [*Data are provided specific to an audio-only podcast rather than…*] with a visual component (e.g. a podcast + lecture slides, a podcast + meditative animation)   The following exclusion criteria were added:   - Audio recordings are music, sound effects or soundscapes alone (e.g. intervention involves participants listening to music) - Studies using a podcast as a control-group only, unless the control group has an ‘active’ component that would theoretically impact on the mental health outcome(s) - The intervention or experiment simply has some kind of audio recording prompt or instruction that is not also described or contextualised as a podcast - The intervention or experiment involves participants creating or producing a podcast, rather than listening to a podcast |
| Outcomes | - Additional outcome domains were added that were not initially flagged, adding chronic pain, stress and carer burden - Added text to clarify that primary and secondary outcome variables would be eligible |
| Evidence selection | The following text was added to provide more detail about the search and decision-making process, particularly for grey literature.   - Systematic reviews: Systematic reviews around relevant topics will be read to identify any relevant articles included in the review. For example, if a systematic review is around app-based interventions and mentions that podcasts are included, its reference list will be scanned for any relevant articles. - Unavailable papers, or papers missing results or information: Authors may be contacted to request further information if required, such as missing results or information about methodology. Authors will be contacted a maximum of two times via email; if they do not respond to a follow up email within a month, no further contact will be made. - Conference proceedings and dissertations: The reviewers will check if any posters, slides, reports or publications from same study are published, and download these if available. Authors may be contacted as above. If no further publications are available, the abstract or dissertation may be used as the only unit of analysis and extracted. - Clinical trial registrations: If a study is complete, the reviewers will check for any attached data or any clinical study reports, conference proceedings, reports or publications are available from the same study.  If study is not complete or no publications are yet available, it will be marked as an ‘ongoing study’ and saved to a ‘Characteristics of ongoing studies’ table; the reviewers will check again if more than 12 months pass between the initial search and publication. Authors may be contacted as above.   Reference lists of any journal articles included in the final systematic review will also be searched, and screened using the same systematic process in Covidence. These reference lists will be saved in Web of Science and uploaded to Covidence. The reference lists of dissertations will be subject to title screening; any titles deemed relevant will be downloaded and screened systematically in Covidence. Any other papers identified that seem relevant will also be screened. |

1. These diagnoses were chosen as they are aligned with the broad organisational focus of SANE, one of the key collaborators of the study. [↑](#footnote-ref-1)
2. Includes grey literature: conference proceedings [↑](#footnote-ref-2)
3. Includes grey literature: conference proceedings [↑](#footnote-ref-3)
4. Includes grey literature: dissertations and theses [↑](#footnote-ref-4)
